# Infodemic Versus Viral Information Spread: Key Differences and Open Challenges

**DOI:** 10.2196/57455

**Published:** 2025-05-07

**Authors:** Matteo Cinelli, Francesco Gesualdo

**Affiliations:** 1 Department of Computer Science, Sapienza University of Rome, Via Regina Elena 295, Rome, 00100, Italy, 39 3397898012; 2 Department of Translational Research and New Technologies in Medicine and Surgery, University of Pisa, Pisa, Italy

**Keywords:** infodemic, information spreading, infodemiology, misinformation, artificial intelligence, information virality, public health, multidisciplinary, data science, AI, difference, challenge

## Abstract

As we move beyond the COVID-19 pandemic, the risk of future infodemics remains significant, driven by emerging health crises and the increasing influence of artificial intelligence in the information ecosystem. During periods of apparent stability, proactive efforts to advance infodemiology are essential for enhancing preparedness and improving public health outcomes. This requires a thorough examination of the foundations of this evolving discipline, particularly in understanding how to accurately identify an infodemic at the appropriate time and scale, and how to distinguish it from other processes of viral information spread, both within and outside the realm of public health. In this paper, we integrate expertise from data science and public health to examine the key differences between information production during an infodemic and viral information spread. We explore both clear and subtle distinctions, including context and contingency (ie, the association of an infodemic and viral information spread with a health crisis); information dynamics in terms of volume, spread, and predictability; the role of misinformation and information voids; societal impact; and mitigation strategies. By analyzing these differences, we highlight challenges and open questions. These include whether an infodemic is solely associated with pandemics or whether it could arise from other health emergencies; if infodemics are limited to health-related issues or if they could emerge from crises initially unrelated to health (like climate events); and whether infodemics are exclusively global phenomena or if they can occur on national or local scales. Finally, we propose directions for future quantitative research to help the scientific community more robustly differentiate between these phenomena and develop tailored management strategies.

## Introduction

The definition of infodemic has evolved over the years. It started from being an “epidemic of information,” as defined by Rothkopf in 2003 [1] in the context of the severe acute respiratory syndrome (SARS) outbreak, to then include the element of misinformation [2-4], especially when the concept gained momentum during the COVID-19 pandemic. Since then, thanks to the efforts of the World Health Organization (WHO) and collaboration with academics, infodemiology, that is, “the science of distribution and determinants of information in an electronic medium, specifically the Internet, or in a population, with the ultimate aim to inform public health and public policy” [2,5], has evolved from a primarily descriptive discipline into a more comprehensive field [6]. This transformation has integrated insights from behavioral, medical, and complex systems sciences, leading to a more comprehensive understanding that encompasses a broader range of phenomena and challenges.

From the earliest WHO reports on the COVID-19 infodemic and the global technical consultation of April 2020 [7], the infodemic was defined as “an overabundance of information—some accurate and some not—that occurs during an epidemic.” Later definitions introduced the concept of information overflow [8], emphasizing the difficulty individuals face in identifying trustworthy information during crises. This broader conceptualization acknowledges that an infodemic extends beyond misinformation, as it is shaped by public concerns, information voids, and digital transformation processes that amplify content circulation.

Recent discussions, including those from the Fifth WHO Infodemic Management Conference [9], have further refined this perspective, by highlighting the layered and multifaceted nature of infodemics. The conference underscored how infodemics emerge from a confluence of structural, behavioral, and technological factors, including the characteristics of the information ecosystem, the role of digital platforms in amplifying or mitigating information spread, the psychological and social drivers of user engagement, and the dimension of trust in public institutions.

As digital platforms continue to evolve, new technological advancements further complicate the landscape: after the COVID-19 pandemic, the release of large language models to the general public raised discussions on the potential role of generative artificial intelligence in fueling future infodemics [10], adding another dimension to the challenge of managing information flows during crises.

While acknowledging this complexity, in this paper, we focus on one specific dimension of the infodemic phenomenon: the quantification of information production. By narrowing our analysis to this aspect, we aim to clarify how excess information during an infodemic differs from a general viral information spread. A more precise characterization of the quantitative aspects of information overabundance can help differentiate infodemics from other forms of information dissemination, guiding efforts to improve detection methods and to ensure that public health strategies can respond to crises in an even more informed manner.

The concept of an overabundance or excess of information, which underpins the current definition of infodemic, could be better described quantitatively by focusing on different aspects of information production and circulation. First, to our knowledge, there is broad recognition that individuals are continuously exposed to vast amounts of information, raising the question of how to benchmark overabundance. In other words, should it be measured with respect to content production regarding different topics of discourse or with respect to the same topic (assuming it was present in the debate) in a previous time window? Additionally, are different time windows comparable on social media, given the high turnover of users within digital communities? Second, should a spike in interest or a heated public discourse about a health-related issue happening on the web automatically be labeled as an infodemic or can it “just” be considered a noteworthy episode? And, further, can we provide estimates of the minimum lasting time for information overabundance to become an infodemic? In this scenario, we believe that understanding the differences between excess information during an infodemic and information virality can be useful to advance our understanding of infodemic processes. This distinction can inform the development of more precise indicators for enhanced infoveillance systems, enabling better monitoring and intervention strategies.

For these reasons, in what follows, we will list a set of key points that, building upon recent literature, differentiate excess information during an infodemic from a viral process of information diffusion and that could be turned, in certain instances, into viable measurements, with the aim of pointing towards new research directions. Instrumental to our purpose is to clarify what we mean by a viral process of information spread: a viral information spread or virality refers to the rapid and widespread dissemination of information or content, often through social networks and web-based platforms [11]. It follows that a viral process also generates an “overabundance” of information that, in theory, should be distinguishable from that generated by an infodemic in many respects.

## Differences Between an Infodemic and a Viral Information Spread

**Figure 1.**
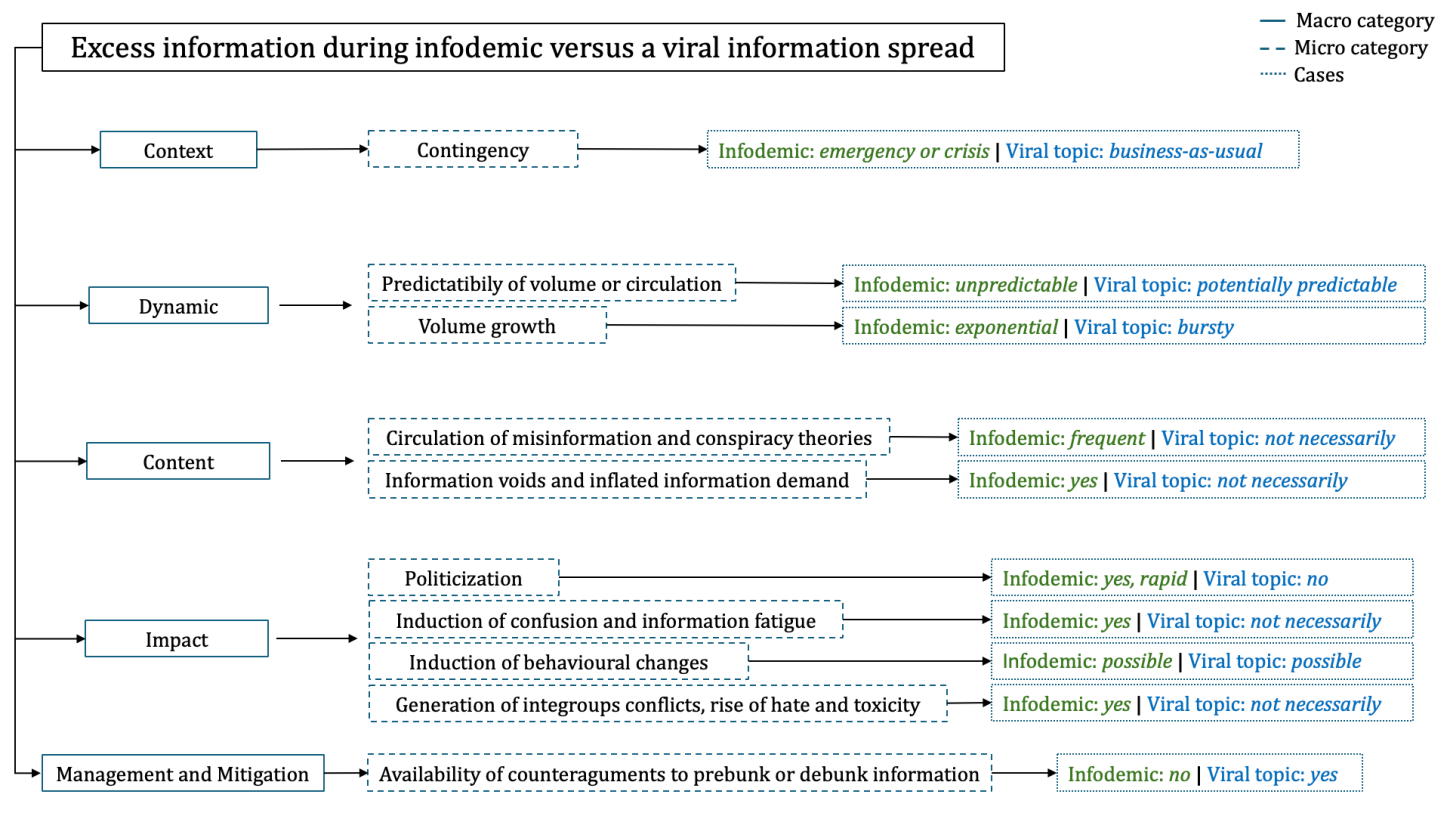
Summary of differences between excess information during an infodemic and a viral information spread.

In this section, we list a set of differences between the excess information observed during an infodemic and a viral information spread, considering up to 5 characterizing dimensions used for classification. Categories and differences are presented in a conceptual map in Figure 1.

### Context and Contingency

An infodemic is associated with a (health) emergency or crisis, occurring in contexts where there is a heightened need for timely information and leading to an increased need for information dissemination to the public. In contrast, a viral topic may emerge during periods of “business-as-usual,” meaning it does not necessarily coincide with any crisis or emergency. The timing and context of these events are crucial for differentiating an infodemic from a viral topic, with infodemics being crisis-driven and viral topics being more routine. However, in some cases, the initial magnitude of a health crisis may not be immediately clear, making it difficult to distinguish between a transient viral circulation of information and an evolving infodemic that could escalate in magnitude and impact.

### Dynamic: Volume Growth and Predictability

The dynamics of how information spreads during infodemics differ significantly from those of viral topics. The production of information during infodemics is characterized by its unpredictable volume and spread. Content volume often grows exponentially during an infodemic and remains steady over extended periods. On the other hand, specific topics characterized by a certain seasonality or scheduled events of great resonance (eg, elections), that fall into the definition of virality, tend to have more predictable patterns. A similar reasoning holds for topics displaying sublinear, superlinear, or wavy behaviors in their volume growth, whose evolution, unlike in the case of infodemics, can be inferred by precise mathematical models [12,13]. However, any output by a forecasting model would require some initial input data, meaning that no predictions can be made until some pieces of content are released. It is also worth reminding that any expectation or prediction related to events in the social sphere carries a degree of uncertainty that depends on the contingency of exogenous events. Such uncertainty can lead to significant fluctuations in the phenomenon being studied, potentially rendering model predictions unreliable. This is a fundamental characteristic of complex systems and a key challenge in studying a constantly evolving society.

An example of the differences between viral topics and infodemics can be observed by comparing 4 cases or events that have gained substantial collective attention, namely the COVID-19 pandemic, the Russo-Ukrainian conflict, the death of Queen Elizabeth II, and Christmas. To illustrate this, and under the hypothesis that search interest in a topic correlates with content production [14], we used Google Trends data [15], which provide weekly time series of worldwide searches on Google over a 5-year period. We analyzed the search volumes for the following topics: “COVID-19,” “Queen Elizabeth,” “Ukraine,” and “Christmas,” though similar results were obtained when substituting “Russia” for “Ukraine.” The weekly search volumes for these 4 terms were normalized on a scale of 0 to 100, where 100 represents the highest recorded search volume across all 4 terms, and 50 indicates half of that peak. A score of 0 indicates insufficient data.

**Figure 2.**
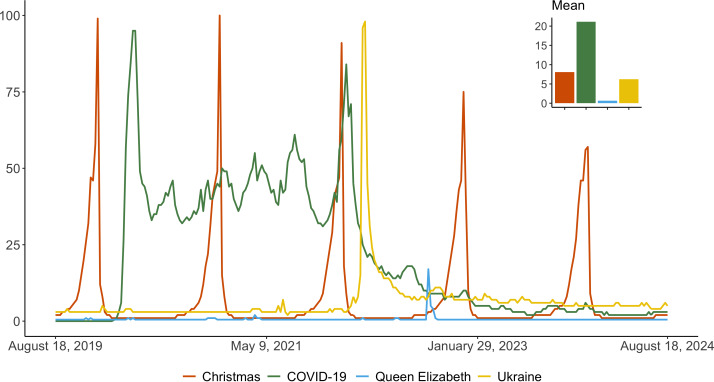
Time series of relevant topics including 1-time virality (Queen Elizabeth), seasonal virality (Christmas), infodemic (COVID-19), and infodemic-like (Ukraine). Data were obtained by searching keywords on Google Trends. The inset shows the average value of normalized searches for each keyword.

From Figure 2, we can observe a clear difference between the 4 terms. While the death of Queen Elizabeth II gathered significant attention and became a viral event, its trajectory is markedly different from the other cases. COVID-19 shows a trend that takes about 6 weeks to peak, after which it continues to receive a high volume of searches over an extended period. The Russo-Ukrainian conflict presents a different pattern, falling somewhere in between the other 2 cases. Search volume peaks within approximately 3 weeks, followed by a decline to much lower, though still notable, levels—perhaps suggestive of an infodemic (note also that the situation in Ukraine is listed among the WHO’s health emergencies [16]). Lastly, the case of Christmas serves as an example of a seasonal and thus predictable topic, which still falls within the definition of virality.

### Content: Circulation of Misinformation and Information Voids

Content-wise, infodemics are frequently associated with a flood of mis- or disinformation and conspiracy theories, which is not necessarily the case with viral topics. During infodemics, the increased production of content co-occurs with the presence of information voids and inflated information demand [17,18], where gaps in credible information contribute to the uncontrolled spread of both accurate and misleading content. In contrast, viral topics do not necessarily involve such voids or demands, and the spread of misinformation may not be as prevalent.

### Impact: Politicization, Confusion, Behavioral Changes, Social Cohesion, and Toxicity

The impact of an infodemic can be far-reaching. Infodemics tend to become rapidly politicized [19,20], with misinformation being shared and consumed as it aligns with the views of specific social and political groups. This rapid politicization is less likely with viral topics. Furthermore, infodemics can induce confusion and information fatigue [21-23] (sometimes referred to as information overload or infoxication), contributing to significant behavioral changes among individuals [24]. Viral topics, while they may also influence behavior [25], do not necessarily lead to such widespread confusion or fatigue.

An infodemic can erode social cohesion [26] by generating intergroup conflicts and increasing levels of toxicity in the public discourse [27]. This is a significant consequence of infodemics, fueled by the spread of divisive misinformation. Viral topics, while they may stir debate, do not typically lead to the same level of social disruption or rise in toxicity. At present, no systematic research has examined the correlation between topic virality and toxic speech usage. However, in the case of infodemics, researchers have observed a consistent association with toxic speech, starting with Sinophobia at the onset of the COVID-19 pandemic [27] and continuing with heated debates over vaccines in later stages [28], whereas in the case of viral topics, such occurrences have been limited to only a few instances [29,30].

### Management and Mitigation: Availability of Counterarguments

When it comes to managing and mitigating the effects of infodemics and viral topics, a stark contrast emerges. During an infodemic, there is often a lack of established counterarguments, making it challenging to pre- or debunk misinformation [20]. In such situations, the scientific community may need to rely heavily on the best evidence available at the time due to the absence of consensus statements or previously established facts. On the other hand, for viral topics related to public health, such as vaccines, there is often an abundance of counterarguments from past debates that can be employed to address mis- or disinformation. These resources can also be effective in crafting prebunking strategies.

For instance, in recent years, a decline in coverage rates for the human papillomavirus (HPV) vaccine has been documented in Ireland [31], Denmark [32,33] and Colombia [34], primarily due to the viral circulation of web-based and social media content questioning the vaccine’s safety profile. While one could argue that the spread of misinformation in these cases can be described as an infodemic, this characterization remains debatable due to insufficient data for measuring information overabundance in digital and physical environments. Instead, it can be more accurately hypothesized that these incidents represent a viral spread of misinformation. In some cases, the impact of this misinformation has been long-lasting. However, in most instances, the availability of robust safety data on the HPV vaccine has enabled public health agencies to conduct communication campaigns that effectively restored public confidence in the HPV vaccine. By contrast, during the COVID-19 pandemic, public health agencies faced a tougher challenge in countering vaccine hesitancy, as they initially lacked real-world safety data to support their efforts.

## Discussion

### Open Points

As a concluding step, moved by the reasoning on the difference between excess information in an infodemic and a viral information spread, we discuss certain open points regarding the infodemic phenomenon.

Is an infodemic solely associated with a pandemic? Not necessarily; other health emergencies, as in the case of abortion [35], may generate an infodemic. Furthermore, health emergencies can arise from various factors beyond the spread of communicable diseases. For instance, the WHO includes events such as conflicts in Sudan and the neighboring countries [36] and other health and humanitarian crises [37] in the list of present and past health emergencies.

Is an infodemic limited to health-related issues? It is probable that future infodemics will arise during crises initially unrelated to health. For example, the increasing likelihood of extreme climate events over time may trigger prolonged (infodemic-like) debates fueled by various factors such as disasters or government interventions. In such cases, as crises are likely to touch every societal layer and health emergencies are deeply interconnected with socioeconomic and environmental factors, the health community would be anyway involved. Recent examples include the Israeli–Palestinian and the Russo-Ukrainian conflicts. On a related note, an infodemic might have also occurred following the launch of ChatGPT, an event that engaged a broad audience in the artificial intelligence debate, raising concerns and causing confusion about the role of this new technology in our future.

Is the infodemic exclusively a global phenomenon? Given that health emergencies and topics of public concern are not necessarily international (ie, they are not public health emergencies of international concern), an infodemic may occur at a national level or even at a more local scale. For instance, not all countries may be subject to an overabundance of information at the same time, switching in turn from an infodemic regime to a noninfodemic one. An example regarding the limited geographical reach of an infodemic may be represented by the previously mentioned case of the contagion of psychogenic reactions and the consequent HPV vaccine drop in Colombia due to the spread of videos, published on social media platforms, documenting false adverse reactions post vaccination [34].

### Conclusion

In this paper, we have established a list of key differences that distinguish the excess information production during an infodemic from viral episodes of information diffusion. One key point is that the primary distinctions often become apparent in the medium to long term, including the overabundance of information, the protracted virality over time, the confusion of individuals, and the clear consequences for society. It follows that, in the shorter period, the two phenomena may appear somewhat indistinguishable, most likely as in the case of the transition from an epidemic to a pandemic. However, considering the vast amount of data and early signals potentially detectable on web-based and social media platforms, a significant phenomenon such as an infodemic cannot be regarded as something one realizes is happening as it occurs. This conceptual work aims to contribute to a better understanding of infodemics and information virality and may serve as a baseline for developing improved measures of information overabundance to support the multilayered process of infoveillance. In practice, possible efforts to measure the circulation of information during an infodemic could include developing causal models of infodemic evolution based on a series of independent variables, both epidemiological and nonepidemiological, to understand how non–health-related factors contribute to its progression. Establishing and utilizing appropriate baseline models to measure information overabundance across topics of similar and different natures would also be crucial. Additionally, leveraging the wide availability of viral web-based phenomena to create a set of case studies could help to compare the growth rates characterizing the initial waves of information diffusion of a new potential infodemic, thus providing reliable early warnings. While this may not be a primary concern for public health, it is valuable to highlight how different information-spreading processes may share certain characteristics (eg, overabundance) with the infodemic. Recognizing these commonalities could allow research to incorporate insights and practices developed in the study of infodemics into other contexts and vice versa, thus enhancing an always relevant multidisciplinary debate around this domain of knowledge. Future research efforts could shift the focus on how the dynamics of information consumption differ in infodemic versus viral contexts, as health-related content tends to have even less impact on behavior when individuals are not experiencing a personal, family, or social event that alters their need for information.

Finally, beyond serving as a checklist for infodemic recognition, the listed differences account for observations in the data regarding the COVID-19 infodemic and other topics, providing a challenging perspective that the public health community could employ to enhance existing infodemic management frameworks.

## Abbreviations

HPV: human papillomavirus
SARS: severe acute respiratory syndrome
WHO: World Health Organization
